# Post-traumatic stress symptoms and benefit finding: a longitudinal study among Italian health workers during the COVID-19 pandemic

**DOI:** 10.1007/s00127-023-02475-3

**Published:** 2023-04-08

**Authors:** Luca Negri, Marta Bassi, Roberto Accardi, Antonella Delle Fave

**Affiliations:** 1 Department of Pathophysiology and Transplantation, Università degli Studi di Milano, Via F. Sforza 35, 20122 Milan, Italy; 2 Department of Biomedical and Clinical Sciences, Università degli Studi di Milano, Milan, Italy; 3 Health Professions Directorate, Fondazione I.R.C.C.S. Ca’ Granda Ospedale Maggiore Policlinico, Milan, Italy

**Keywords:** Benefit finding, PTSD, Health workers, COVID-19, Moderation, Longitudinal

## Abstract

**Purpose:**

Research has highlighted that the exposure of healthcare professionals to the COVID-19 pandemic for over two years can lead to the development and persistence of symptoms characteristic of Post-Traumatic Stress Disorder (PTSD), with serious consequences on both the individual well-being and the quality of care provided. The present study was aimed at investigating the role of benefit finding in moderating post-traumatic stress symptoms (PTSS) over time.

**Methods:**

The longitudinal study, conducted between April and October 2020, involved 226 Italian health workers (44.7% nurses and midwives, 35% doctors, 20.3% technical and rehabilitation professionals), who filled out an online survey at the beginning of the study (T_1_), after three months (T_2_), and after six months (T_3_).

Participants (77.4% women; mean age = 41.93, SD = 12.06) completed the PTSD Checklist for DSM-5 (PCL-5) and Benefit Finding, a 17-item questionnaire measuring the perceived level of positive consequences derived from stressful experiences. A hierarchical regression analysis highlighted the moderating effect of benefit finding (T_2_) on the association between PTSS values at T_1_ and T_3_.

**Results:**

A buffering effect was observed, with higher benefit finding levels reducing the magnitude of the bivariate association between PTSS assessed at the beginning and at the end of the study.

**Conclusion:**

Findings suggest the potential mental health related benefits of interventions allowing health professionals to identify positive aspects in the experience of working under prolonged emergency circumstances, such as the pandemic ones.

## Introduction

Italy was the first European country to be strongly hit by the SARS-CoV-2 outbreak. The first national lockdown was declared on March 8 and lasted until May 3, 2020; a gradual decrease in COVID-19 cases was observed between May and mid-August, followed by a growing incidence trend and a renewed outbreak in October and November 2020 [1].

Worldwide, several studies explored mental health disorders during the pandemic in the general population; results showed a high prevalence of Post-Traumatic Stress Symptoms (PTSS), and Post-Traumatic Stress Disorder (PTSD), together with anxiety and depression [2–8]. Similar results were found among healthcare workers (HWs) in different countries [9–13]. Especially during the first and sudden outbreak of the pandemic, HWs experienced remarkable strain in their frontline role, due to dearth of protective equipment, exposure to high infection risk, and lack of available treatment protocols for assisting patients infected by Sars-Cov-2, especially when the disease progression led to critical conditions. Not surprisingly, several studies conducted across countries identified high levels of PTSS among HWs [14–16]. As concerns Italian HWs, in a survey conducted during the first pandemic outbreak almost half of the participants reported presence of PTSS [17]; in another work, 26.2% of the sample met the criteria for a provisional PTSD diagnosis [18].

According to the International Classification of Diseases (ICD-11) [19], PTSD development is strongly associated with exposure to a trauma, defined as an extremely threatening or horrific event or series of events. Similarly, the current diagnostic and statistical manual of mental disorders (DSM-5) defines PTSD as a psychological condition that can occur after exposure to a potentially traumatic event [20]. As highlighted in recent works, the COVID-19 pandemic and associated consequences could be considered as a potentially traumatic event in both classification systems [21, 22]. According to the DSM-5, a PTSD diagnosis requires the presence of PTSS including re-experiencing the traumatic event, avoidance of related stimuli, intrusive behaviors, and worsening cognition and mood after the traumatic event. A large body of research showed that PTSS and PTSD are associated with general distress and a higher risk of developing both physical and mental co-morbidities [23–25].

From a complementary perspective, an increasing number of studies have been focused on the individual and environmental resources that can be mobilized or developed to cope with or adapt to stressful events and potentially life-threating conditions [26–31]. Among these resources, benefit finding (BF) is defined as the identification of benefits from adversities. Benefits can be detected at the individual, relational and transcendent levels; they include, for example, increased appreciation for life, awareness of deepened interpersonal relationships, enhanced sense of spirituality, and life purpose [32–36]. Benefit finding has been associated with optimal adaptation to stressful situations, including pandemics [37, 38]. Both perception of and active search for benefits deriving from stressful events have been accounted for as cognitive meaning-making reappraisal strategies promoting individual well-being [39–41].

The identification and acknowledgement of positive contributions from traumatic experiences are an important resource in coping with life-threating situations [42, 43]. It is also a core component of Post-Traumatic Growth (PTG) [44, 45], defined as the process allowing individuals who are coping with trauma or life crises to strengthen their perceptions of self, others, and the meaning attributed to the traumatic experience itself [45]. Although similar, PTG and benefit finding differ in terms of both predictors and development patterns [46, 47]. Concerning predictors, Sears and colleagues [47] found a significant longitudinal association between BF and the personal characteristics of optimism and hope; this relationship was not confirmed for PTG. Research works also suggested different timing in the emergence and development of these two processes, with BF potentially starting immediately after the traumatic experience, and PTG requiring more time (i.e. weeks/months/years), due to the associated in-depth restructuring process [43, 47]. Overall, most studies addressing positive contributions following adversities or trauma were focused on PTG and associated dimensions, while only limited attention was devoted to benefit finding; efforts within this research area were mainly aimed at investigating the relationship between BF and adaptation to stress/life-threating events. Results were however controversial: While a significant association was identified in some studies between BF and lower or decreased levels of distress [32], other works did not confirm this relationship [47, 48]. The even fewer studies devoted to the investigation of the moderating role of BF on psychological distress also provided inconsistent results. In some of them, a significant interaction emerged between BF and severity of stressors in predicting improved psychological adjustment among disaster survivors [49] and women living with HIV/AIDS [50]; BF was also found to buffer the relationship between combat stress and PTSD symptoms among active soldiers following a 15-month deployment, but only under conditions of supportive officer leadership [51]. Opposite patterns were instead detected in other studies, in which the interaction between high levels of BF and breast cancer severity predicted worse mental health outcomes over time [36].

Within the international literature exploring the psychological correlates of the COVID-19 pandemic, only a limited amount of studies was aimed at investigating PTG and BF, both in the general population and among HWs. Results from a large-scale Chinese survey involving nurses showed that 39% of participants experienced PTG; further, nurses working at COVID-19 hospitals and caring for patients in critical care units reported higher PTG scores than nurses not engaged in these services [52].

As for BF, results from the general population showed that mortality concerns related to COVID-19 were cross-sectionally associated with higher BF values; BF was, in turn, positively correlated with life satisfaction, meaning in life, self-esteem, and resilience, while negatively correlated with levels of depression and stress [53].

BF was also found to longitudinally mediate the relationship between support seeking and well-being among Chinese participants from the general population during the COVID-19 pandemic [37].

To the best of our knowledge, no longitudinal studies were yet conducted to jointly monitor PTSS and BF during the COVID-19 pandemic in a population specifically exposed to the related strain and traumatic experiences, such as health professionals. In particular, no longitudinal studies were conducted to investigate the moderating role of benefit finding on PTSS development among these workers.

### Study aims

The current study was aimed at investigating: (a) PTSS and BF development among Italian HWs actively working during the COVID-19 pandemic, and (b) to test the moderating role of BF on PTSS development across time. Due to the unpredictable development of the COVID-19 pandemic, no a priori hypotheses were formulated for the first aim (a); concerning the second aim (b), the identification of benefits from adversities related to the COVID-19 pandemic was hypothesized to buffer the magnitude of the association between participants’ PTSS measured at the beginning and at the end of the longitudinal study.

## Method

### Procedures and participants

The present study was conducted between April and October 2020, and consisted in three consecutive waves of data collection: April 15 to May 15 (T_1_), July 6 to August 6 (T_2_), and October 14 to October 30 (T_3_). Data on levels of PTSS were collected at T_1_, T_2_, and T_3_, while perceived BF was assessed at T_2_ and T_3_. Participants were HWs actively working in Lombardy, the Italian region in which the highest rates of contagion and deaths were recorded during the first pandemic outbreak [54, 55]. Potential participants were contacted through an e-mail message containing detailed information on the study features and the link to the online questionnaires, which was sent to HW associations in Lombardy with the invitation to forward it to their members. Both acknowledgment and signature of the informed consent form were mandatory to access the questionnaires. Anonymity was granted by inviting participants to create an individual alphanumeric code, to be used across the three assessment waves.

### Measures

All participants completed two self-reported measures:

*The Post-Traumatic Stress Disorder Checklist for DSM-5* (PCL-5) [56, 57], a 20-item measure assessing PTSS severity experienced over the previous month on scales ranging from 0 ‘not at all’ to 4 ‘extremely’. A provisional diagnosis of PTSD can also be formulated when the PCL-5 score is ≥ 33 [57]. The PCL-5 was completed at T_1_, T_2_ and T_3_.

*Benefit Finding* (BF) [32, 36], a 17-item measure assessing the perception of positive contributions to one’s life deriving from stressful and life-threatening experiences. Answers are provided through a 1 ‘I disagree a lot’ to 5 ‘I agree a lot’ scale; higher scores reflect higher perceived benefit; BF was assessed at T_2_ and T_3_.

Socio-demographic and work-related data included age, gender, profession and job seniority; in addition, at T_2_ and T_3_ participants were also asked to report if they had been assigned to a COVID-19 department since T_1_.

### Data analysis

As a first step, descriptive statistics of the study measures were calculated. Cochran’ Q was employed to longitudinally assess changes, across time, in the frequency of participants meeting criteria for a provisional PTSD diagnosis; differences in PTSS scores at T_1_, T_2_, and T_3_ were tested using a repeated measures ANOVA followed by post-hoc comparisons with Bonferroni correction; BF levels (T_2_, T_3_) were also compared using a Student *t*-test for paired samples. Data were checked for repeated measures ANOVA and Student *t*-test assumption violations; namely, presence of extreme outliers together with normality assumption were inspected for both analyses; the ANOVA assumption of sphericity was also tested through Mauchly’s test.

Subsequently, Pearson correlations were calculated to assess the magnitude of associations between PTSS and BF both cross-sectionally and at different time points. Regardless of statistical significance, only values ≥|0.30| were considered as meaningful. Values between |0.30| and |0.49| were interpreted as indices of low correlation, values between |0.50| and |0.69| as indices of moderate correlation, and values ≥|0.70| as indices of high correlation between variables [58].

A hierarchical linear regression analysis using Ordinary Least Square (OLS) estimation technique was performed to test for the direct effects of PTSS (T_1_) and BF (T_2_) on PTSS (T_3_), as well as the moderating effect of BF (T_2_) on the relationship between PTSS at T_1_ and T_3_. Demographic and job-specific dimensions were entered in the regression model as control variables. Data were checked for violations of regression assumptions. In particular, linearity and normality of residuals were investigated through comparison of residual vs fitted values and Q-Q plots visual inspection, respectively; the absence of autocorrelation in residuals was assessed using the Durbin-Watson test while the Breusch-Pagan test investigated heteroscedasticity; as final steps, variance inflation factor (VIF) and leverages inspection were employed to detect multicollinearity issues and influential data points, respectively. Categorical variables with three or more levels were dummy coded; a reference category was selected and employed. Significance of regression coefficients was estimated through 95% confidence intervals (CI) from 2,000 bootstrapped samples. Simple slopes were calculated for the 16th, 50th, and 84th percentile of the distribution, as suggested by Hayes [59]. All slopes were then plotted and tested for significance through Student *t*-test.

In order to assess the reliability of statistically significant results, observed effect sizes were compared with threshold reliability values calculated through sensitivity analysis [60]; f and f^2^ indices were employed to evaluate reliability of ANOVA and regression analyses, respectively [61].

## Results

### Attrition rate and data handling

A total of 721 HWs completed the longitudinal study at T_1_, 357 (49.5%) at T_2_ and 232 at T_3_ (32.2%). Demographic features and distress levels were compared between participants who completed all three data waves and those who dropped out after T_1_ or T_2_. Attrition analysis results indicated that there were no significant differences in age, [*t*(719) = 1.911; *p* = 0.056], gender [χ^2^(1) = 2.36; *p*_Fisher_ = 0.148], job seniority [*t*(719) = 1.40; *p* = 0.162], profession [χ^2^(2) = 0.23; *p* = 0.890], PTSS(T_1_) score [*t*(719) = -0.58; *p* = 0.562], or percentage of provisional PTSD diagnosis at T_1_ [χ^2^(1) = 0.07; *p*_Fisher_ = 0.807].

Out of the 232 participants completing the three waves, four (1.7%) were excluded from the final sample due to missing answers. Moreover, data from two participants (0.86%) were excluded from analyses because they retired from work between T_2_ and T_3_.

### Descriptive statistics

The sample’s demographic and job characteristics are reported in Table 1. Most participants were women, in their forties and working as nurses or midwives; during the overall study period, over 60% of the HWs worked in a COVID-19 department.

**Table 1:** Participants’ demographic and job characteristics

N = 226	N	%	M	SD
Age			41.93	12.06
Gender				
Woman	175	77.4		
Profession				
Medical doctor	79	35		
Nurse/midwife	101	44.7		
Technical and rehabilitation professional	46	20.3		
Job seniority (years)			17.19	12.23
Being assigned to a COVID-19 department				
Before or at T_2_	142	62.8		
At T_3_	80	35.4		

*N* number of participants

Descriptive statistics are reported in Table 2, together with Cronbach α reliability indices and percentage of participants meeting criteria for a provisional PTSD diagnosis. All α coefficients were ≥ 0.90, indicating excellent internal consistency. As for PTSD, almost 40% of HWs received a provisional diagnosis at T_1_.

**Table 2:** Descriptive statistics and Cronbach reliability indices for Post-traumatic stress symptoms and benefit finding

N = 226	T1	T2	T3
PTSS	29.86^a^ (15.52)^b^ [39.8%]^c^ *0.93*^d^	21.29^a^ (14.65)^b^ [20.8%]^c^ 0.*93*^d^	23.03^a^ (14.98)^b^ [24.3%]^c^ *0.94*^d^
BF	–	3.41^a^ (0.62)^b^ 0.*90*^d^	3.42^a^ (0.61)^b^ 0.*90*^d^

*N* number of participants, *PTSS* Post-traumatic stress symptoms, *BF* Benefit finding;

^a^Mean

^b^Standard deviation

^c^Percentage of participants meeting criteria for a provisional Post-traumatic stress disorder diagnosis (PCL-5 ≥ 33)

^d^Cronbach α coefficient

Cochran’ Q was employed to assess differences in the frequency of PTSD diagnosis across time; a significant difference was detected (Q_(2)_ = 39.22; *p* < 0.001). Post-hoc Dunn’s tests with Bonferroni correction showed a higher percentage of PTSD diagnoses at T_1_ than T_2_ (*p* < 0.001), and T_3_ (*p* < 0.001); no differences were found between T_2_ and T_3_ (*p* = 0.82).

Since a violation of sphericity assumption was observed for repeated one-way ANOVA testing differences in PTSS scores at T_1_, T_2_, and T_3_ (W = 0.953; *p* = 0.004), robust estimation techniques based on trimmed mean (20%) and bootstrap sampling (N = 2000) were employed to corroborate results of parametric tests. Welch-James (WJ) statistic [62] was calculated and used for robust hypothesis testing.

Parametric repeated one-way ANOVA resulted in a significant omnibus test (F_(2, 450)_ = 62.36; *p* < 0.001), with an effect size (f = 0.52) higher than the sensitivity threshold (f = 0.21; *N* = 226; 1-β = 0.80; α = 0.05). Pairwise post-hoc comparisons (Bonferroni adjustments) highlighted differences between T_1_ and T_2_ (*p* < 0.001), and between T_1_ and T_3_ (*p* < 0.001). No differences between T_2_ and T_3_ were observed for PTSS (*p* = 0.05). The robust alternative confirmed parametric results: The omnibus test was significant (WJ_(2, 269.9)_ = 20.47, *p* < 0.001) with pairwise comparisons resulting in both a significant T_1_ vs T_2_ (WJ_(2, 269.3)_ = 37.49; 95%CI: [0.41; 0.82]) and T_1_ vs T_3_ (WJ_(2, 269.3)_ = 23.22; 95%CI: [0.28; 0.69]) difference; no difference was instead observed between T_2_ and T_3_ (WJ_(2, 270)_ = 1.83; 95%CI: [− 0.34; 0.07]).

All assumptions were met for the paired Student *t*-test comparing BF at T_2_ and T_3_; no difference was observed between HWs’ means at these two time points (*t*_(225)_ =  − 0.56; *p* = 0.58).

Table 3 shows correlations among study measures; PTSS at T_1_, T_2_, and T_3_ were all positively correlated, with values > 0.60; a high positive correlation was also observed between BF at T_2_ and T_3_. All remaining correlations did not reach meaningfulness threshold.

**Table 3:** Correlations among study measures

N = 226	(1)	(2)	(3)	(4)	(5)	(6)	(7)	(8)
(1) PTSS(T_1_)	–							
(2) PTSS(T_2_)	0.67**	–						
(3) PTSS(T_3_)	0.62**	0.73**	–					
(4) BF(T_2_)	− 0.08	− 0.17*	− 0.12	–				
(5) BF(T_3_)	− 0.08	− 0.13	− 0.18**	0.72**	–			
(6) COVID(T_2_)	0.08^a^	0.13^a^	0.13*^a^	− 0.07^a^	− 0.04^a^	–		
(7) Gender	0.06^a^	0.04^a^	0.03^a^	0.10^a^	0.11^a^	− 0.02^b^	–	
(8) Job seniority	− 0.03^a^	− 0.03	− 0.11	− 0.09	− 0.01	− 0.25**^a^	0.04^a^	–
(9) Medical doctor	− 0.06^a^	0.01^a^	0.04^a^	− 0.12^a^	− 0.06^a^	− 0.06^b^	− 0.05^b^	− 0.01^a^
(10) Nurse/midwife	0.15*^a^	0.11^a^	0.11^a^	0.06^a^	− 0.03^a^	0.16*^b^	0.08^b^	− 0.11^a^
(11) Technical and rehabilitation professional	− 0.12^a^	− 0.14*^a^	− 0.19**^a^	0.06^a^	0.04^a^	− 0.27**^b^	− 0.04^b^	0.15*^a^

*N* number of participants, *PTSS* Post-traumatic stress symptoms, *BF* Benefit finding, *COVID(T*_*2*_*)* Being assigned to a COVID-19 department before or at T_2_ (0 = No, 1 = Yes), Gender: Woman = 0, Man = 1; Medical doctor: 0 = No, 1 = Yes; Nurse/midwife: 0 = No, 1 = Yes; Technical and rehabilitation professional: 0 = No, 1 = Yes

*p < .05

**p < .01

^a^point-biserial correlation

^b^phi coefficient of association

To test the moderating effect of BF on PTSS development, a hierarchical regression analysis was employed. PTSS at T_3_ was considered as the criterion variable; PTSS at T_1_ and BF at T_2_ were treated as predictor and moderator, respectively, and they were inserted at step 1 together with the following control variables: participants’ gender, profession (technical and rehabilitation professionals were considered as reference category), job seniority, and being assigned to a COVID-19 department before or at T_2_. The cross product between PTSS at T_1,_ and BF at T_2_ followed in step 2.

Plot inspection excluded violations of assumptions related to linearity and normality of residuals; Durbin-Watson (D-W = 1.89; *p* = 0.36) and Breusch-Pagan (BP = 9.31; *p* = 0.31) tests further excluded any autocorrelation or heteroscedasticity issue, respectively. While no influential data points were detected through leverages inspection, a high level of correlation (VIF values ≥ 10) was observed among variables. To reduce multicollinearity, PTSS at T_1_ and BF at T_2_ were centered at their mean values prior to creating their product term [63]. No multicollinearity issues were detected for the model with centered variables (observed VIF values < 2.5).

Results from hierarchical regression analysis are reported in Table 4.

**Table 4:** Hierarchical regression analysis for PTSS at T_3_

	B	SE	β	95% CI		*F*	DF	Δ*R*^*2*^	*R*^*2*^
				Lower	Upper				
Step 1						22.19	(7, 218)	–	0.42***
Gender	0.07	1.78	0.01	−3.48	3.46				
Medical doctor^a^	4.23	2.21	0.13	−0.46	8.31				
Nurse/midwife^a^	2.87	2.25	0.10	−1.49	7.48				
Job seniority	− 0.09	0.06	−0.07	−0.21	0.03				
COVID(T_2_)	1	1.58	0.03	−2.25	3.98				
PTSS(T_1_)	0.59***	0.05	0.61	0.47	0.70				
BF(T_2_)	−1.62	1.44	−0.07	−4.52	1.20				
Step 2						21.37	(8, 217)	0.02**	0.44***
Gender	0.60	1.75	0.02	−2.81	3.97				
Medical doctor^a^	4.18	2.20	0.13	−0.46	8.26				
Nurse/midwife^a^	2.94	2.21	0.10	−1.45	7.41				
Job seniority	−0.11	0.06	−0.09	−0.24	0.01				
COVID(T_2_)	0.96	1.59	0.03	−2.30	4.03				
PTSS(T_1_)	0.58**	0.05	0.60	0.49	0.69				
BF(T_2_)	−1.93	1.42	−0.08	−4.67	0.96				
PTSS(T_1_) x BF(T_2_)	−0.22**	0.08	−0.16	−0.35	−0.05				

Gender: Woman = 0, Man = 1

^a^dummy coded variables with Technical and rehabilitation professionals as reference category (0 = No, 1 = Yes); COVID(T_2_) = Being assigned to a COVID-19 department before or at T_2_ (0 = No, 1 = Yes)

*PTSS* Post-traumatic stress symptoms, *BF* Benefit finding, *PTSS(T*_*1*_*)* × *BF(T*_*2*_*)* Interaction term, SE = Standard errors and CI = Confidence interval based on 2000 bootstrap samples

**p < 0.01

***p < 0.001

**Fig. 1:**
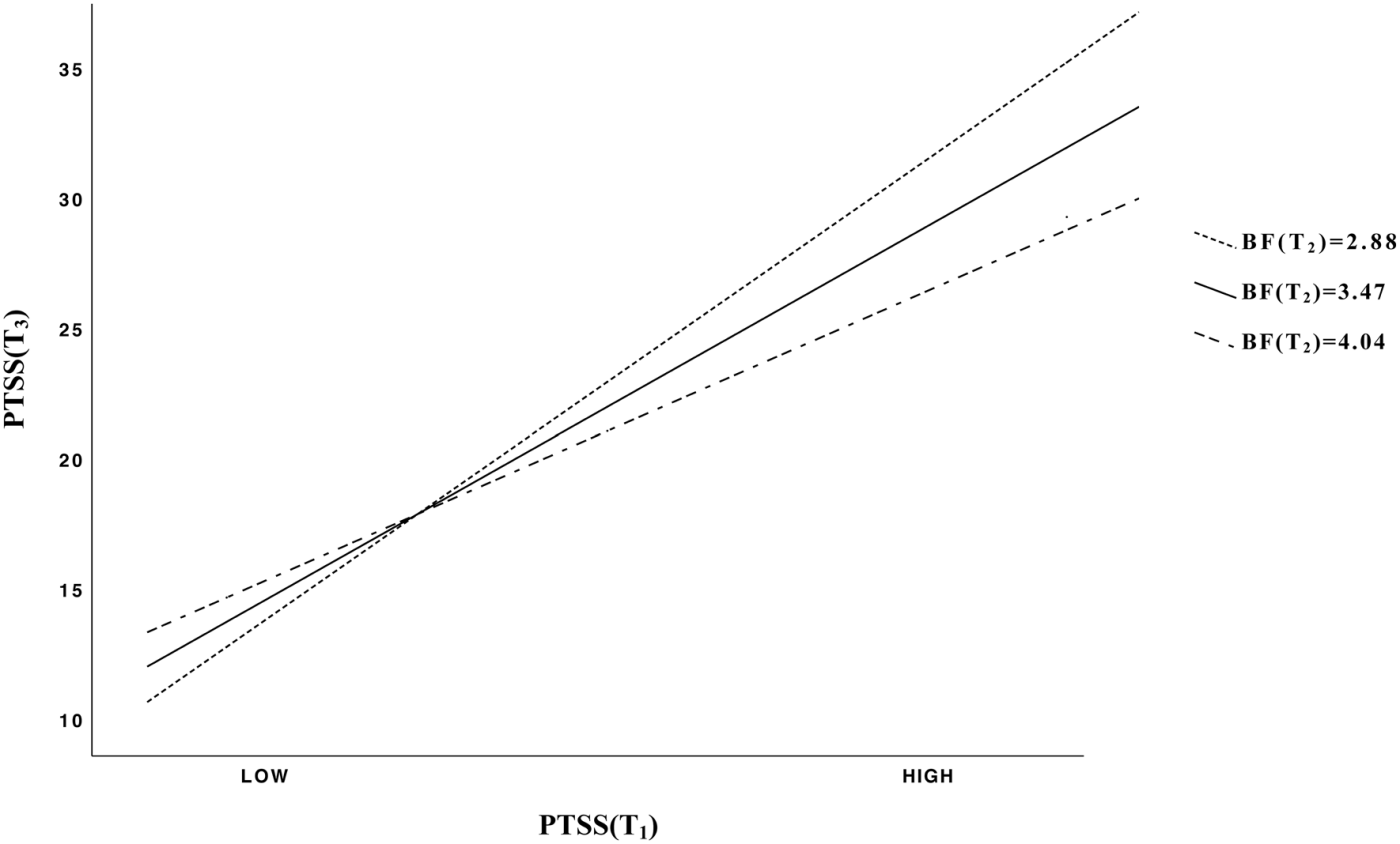
Interaction between post-traumatic stress symptoms at T_1_ and benefit finding at T_2_ on post-traumatic stress symptoms at T_3_

The overall model was significant (F_(8,217)_ = 21.37; *p* < 0.001) and explained 44% of the variance of PTSS at T_3_ with an effect size f^2^ = 0.78 higher than sensitivity threshold (f^2^ = 0.068; *N* = 226; 1-β = 0.80; α = 0.05). A significant positive effect was observed for PTSS at T_1_ (*p* < 0.001), while no linear association was found between BF at T_2_ and PTSS at T_3_; a significant negative effect was instead identified for the product between PTSS at T_1_ and BF at T_2_ (*p* = 0.004). The interaction term accounted for an additional 2% of model variance, supporting the moderation hypothesis; the associated effect size to R^2^ increase was f^2^ = 0.044, higher than sensitivity threshold (f^2^ = 0.035; *N* = 226; 1-β = 0.80; α = 0.05). The interaction was probed by testing the conditional effects of PTSS(T_1_) at three levels of BF(T_2_): 16th (BF = 2.88), 50th (BF = 3.47), and 84th (BF = 4.04) percentile of the distribution [59]. A positive and significant association was detected between PTSS(T_1_) and PTSS(T_3_) for BF(T_2_) at 16th (*t*_(217)_ = 11.22, *p* < 0.001; 95%CI: [0.58; 0.82]), 50th (*t*_(217)_ = 11.30, *p* < 0.001; 95%CI: [0.47; 0.67]), and 84th (*t*_(217)_ = 6.43, *p* < 0.001; 95%CI: [0.30; 0.58]) percentile. The moderation of BF(T_2_) on the relationship between PTSS at T_1_ and at T_3_ is plotted in Fig. 1; a buffering effect of BF at T_2_ was observed, with higher BF(T_2_) levels reducing the magnitude of the bivariate association between PTSS(T_1_) and PTSS(T_3_).

## Discussion

The present study was aimed at investigating post-traumatic stress symptoms (PTSS) and benefit finding (BF) among health workers during the COVID-19 pandemic; the moderating role of BF on PTSS development was also inspected and longitudinally tested.

The first wave (T_1_) took place during the final period of the first national lockdown; not surprisingly, and in line with other studies [17, 64, 65], almost 40% of the participants reported PTSS levels meeting criteria for a provisional PTSD diagnosis. This result attests to the strain and difficulties HWs were exposed to during the pandemic outbreak. Compared to T_1_, a significantly lower percentage of participants met a PTSD provisional diagnosis at T_2_ (July–August 2020); this difference is consistent with the steadily decreasing trend of the infection rate during Summer 2020, corresponding to the T_2_ data collection. Subsequently, however, despite the stable rise in COVID-19 cases observed between August and October 2020 (when the third data collection took place) no significant difference emerged in PTSS values, compared to T_2_. Individual resources such as benefit finding, effortfully mobilized and/or built by health workers during the previous months, in order to fulfill their demanding role in the community, could have played a significant role in buffering the impact of the pandemic on HWs’ mental health. As results show, participants’ benefit finding scores did not differ between T_2_ and T_3_, similarly to what was observed for PTSS values. Considering that no clinical threshold score is available for BF, it is worth noting that HWs’ mean values at both times fell near the central point of the scale with limited standard deviation; this result suggests that most participants were able to derive positive contributions from coping with COVID-19 related difficulties, at least to some extent. The absence of a significant difference between BF values at T_2_ and T_3_ suggests that the “positive teachings” acquired from the pandemic experience had become part of the stable set of HWs’ personal resources, and thus they were not negatively impacted by the increase of contagion rates observed on October 2020.

As for the relationship between PTSS and BF, no meaningful correlations were found within and across time points. The literature addressing this topic is scarce and related findings are controversial; therefore, comparisons can be hardly made. In line with our results, the absence of a significant relationship with stress or life-threating events was observed when BF was longitudinally assessed in patients with early-stage breast cancer [47] and colorectal cancer [48]. On the contrary, a significant negative association emerged in a cross-sectional study involving two groups of patients: participants with cancer, and Systemic Lupus Erythematosus [66]. In the context of the COVID-19 pandemic, a negative association was detected between BF and stress among undergraduate students and adults, but the magnitude level was low (r =  − 0.33) and the study design was cross-sectional [53]. Consistent with our findings, instead, in a longitudinal study conducted among Chinese university students from February to May 2020 [67] no meaningful associations of BF with stress and anxiety were detected.

To get a more detailed understanding of the data collected in the present study, a hierarchical regression analysis was performed to investigate the moderating effect of BF on PTSS development; results were controlled for participants’ gender, profession, job seniority, and involvement in a COVID-19 department before or at T_2_ (July–August 2020). BF at T_2_ was found to exert a significant moderating effect on the relationship between levels of PTSS experienced by HWs at T_1_ (coinciding with the first national lockdown) and at T_3_ (October 2020, characterized by a steady increase of contagion rates). More precisely, a buffering effect was observed, with higher BF levels reducing the magnitude of the bivariate association between PTSS assessed at the beginning and at the end of the study. As results showed, HWs’ PTSS measured at T_3_ increased with PTSS measured at T_1_ for low, medium and high values of BF at T_2_. Although significant, the strength of this relationship was reduced for HWs reporting higher scores of BF at T_2_ as a consequence of the significant interaction.

Overall, evidence from the present study provides support to the role of benefit finding in counterbalancing the post-traumatic stress symptoms experienced by healthcare workers during the COVID-19 pandemic. This finding bears promising implications for interventions aimed at promoting mental health among health professionals facing the COVID-19 pandemic, and other future emergency events [68, 69]. Programs aimed at reducing PTSS through cognitive behavioral therapy (CBT), eye movement desensitization and reprocessing (EMDR), and well-being therapy [70, 71] could be integrated with strategies aimed at actively fostering benefit finding through the support of the related meaning-making process. As suggested by Lechner [72], however, acknowledgment of benefit finding should never be “prescribed” by clinicians or therapists, but instead facilitated through deep examination and mutual sharing. Spontaneous reports of benefits, when recognized and cultivated, can be the starting point of the meaning-making process, potentially promoting greater awareness and, ultimately, higher levels of well-being.

Considering both the high levels of PTSS, and the prevalence of provisional PTSD diagnoses observed among HWs during the COVID-19 pandemic, the support of benefit finding could be particularly useful for both the professionals who did not find any benefit in working during the pandemic, and the workers whose self-generated perception of benefits could be acknowledged and further fostered.

### Limitations, strengths and future directions

To the best of our knowledge, this is the first longitudinal study jointly monitoring PTSS and BF among HWs facing the COVID-19 pandemic, with the aim of testing the effect of benefit finding on post-traumatic stress symptoms development. Taken together, findings underscored the useful role of perceiving positive contributions to one’s life in counterbalancing the negative symptoms derived from prolonged exposure to stressful experiences. Besides these novel contributions, limitations have to be acknowledged as well. First, a high attrition rate was observed along the three study waves, with the majority of participants dropping out between T_1_ and T_2_. Caution must therefore be exerted in generalizing the implications of the findings, even though it is worth noting that participants completing all waves did not differ significantly from dropped out ones for any demographic or psychological variables, and comparable dropout rates were observed in other longitudinal studies conducted during the pandemic [37]. A second limitation concerns the self-report measure employed to formulate provisional PTSD diagnoses; in future studies it should be integrated with DSM-5 based diagnosis issued by clinicians. Finally, cross-cultural differences may exist in perceived benefit while facing difficulties of the COVID-19 pandemic.

Despite limitations, results from this study can offer some hints concerning strategies that could be fruitfully implemented to foster the well-being of health workers exposed to the COVID-19 pandemic related challenges [73]. While results from the present research focused on the first months following the pandemic outbreak, future research should explore the role of benefit finding in adjusting to consequences of the pandemic evolution.

Overall, these findings could also provide some suggestions to clinicians and psychotherapists supporting helping professionals engaged in emergency or rescuing interventions.
